# Efficacy of interventions targeted at physician prescribers of opioids for chronic non-cancer pain: an overview of systematic reviews

**DOI:** 10.1186/s12916-024-03287-1

**Published:** 2024-02-20

**Authors:** Katya Peri, Lucy Honeycutt, Erica Wennberg, Sarah B. Windle, Kristian B. Filion, Genevieve Gore, Irina Kudrina, Elena Paraskevopoulos, Areesha Moiz, Marc O. Martel, Mark J. Eisenberg

**Affiliations:** 1 Lady Davis Institute for Medical Research, Jewish General Hospital, Montreal, QC Canada; 2 Temerty Faculty of Medicine and Institute of Health Policy, Management and Evaluation, Dalla Lana School of Public Health, University of Toronto, Toronto, ON Canada; 3 Department of Epidemiology, Biostatistics and Occupational Health, McGill University, Montreal, QC Canada; 4 Department of Medicine, McGill University, Montreal, QC Canada; 5 Schulich Library of Science and Engineering, McGill University, Montreal, QC Canada; 6 Departments of Family Medicine and of Anesthesia, McGill University, Montreal, QC Canada; 7 Departments of Family Medicine, Royal Ottawa Mental Health Center and Queensway Carleton Hospital, Ottawa, ON Canada; 8 Faculty of Dentistry and Department of Anesthesia, McGill University, Montreal, QC Canada; 9 Division of Cardiology, Jewish General Hospital, Jewish General Hospital, McGill University, 3755 Cote Ste-Catherine Road, Suite H-421, Montreal, QC H3T 1E2 Canada

**Keywords:** Opioid, Overview, Systematic review, Umbrella review, Prescribing

## Abstract

**Background:**

To combat the opioid crisis, interventions targeting the opioid prescribing behaviour of physicians involved in the management of patients with chronic non-cancer pain (CNCP) have been introduced in clinical settings. An integrative synthesis of systematic review evidence is required to better understand the effects of these interventions. Our objective was to synthesize the systematic review evidence on the effect of interventions targeting the behaviours of physician opioid prescribers for CNCP among adults on patient and population health and prescriber behaviour.

**Methods:**

We searched MEDLINE, Embase, and PsycInfo via Ovid; the Cochrane Database of Systematic Reviews; and Epistemonikos. We included systematic reviews that evaluate any type of intervention aimed at impacting opioid prescriber behaviour for adult CNCP in an outpatient setting.

**Results:**

We identified three full texts for our review that contained 68 unique primary studies. The main interventions we evaluated were structured prescriber education (one review) and prescription drug monitoring programmes (PDMPs) (two reviews). Due to the paucity of data available, we could not determine with certainty that education interventions improved outcomes in deprescribing. There is some evidence that PDMPs decrease the number of adverse opioid-related events, increase communication among healthcare workers and patients, modify healthcare practitioners’ approach towards their opioid prescribed patients, and offer more chances for education and counselling.

**Conclusions:**

Our overview explores the possibility of PDMPs as an opioid deprescribing intervention and highlights the need for more high-quality primary research on this topic.

**Supplementary Information:**

The online version contains supplementary material available at 10.1186/s12916-024-03287-1.

## Background

In 2015, the life expectancy of adults in the USA entered a period of sustained decline rivaled only by the First World War and the 1918 influenza pandemic [1]. This decrease was caused by overdoses and suicides related to opioid use [1]. In 2017, the number of opioid-related deaths surpassed that of HIV-AIDS during the peak of the AIDS pandemic [2]. From December 2019 to 2020, over 93,000 Americans died because of drug overdoses, accounting for a 29.4% increase that year [3]. Since 1999, the number of opioid-related deaths has increased five-fold. In total, there have been 500,000 opioid-related deaths since the 1990s [4]. Over-prescription of opioids initially triggered today’s opioid crisis [5].

The relative efficacy of opioids remains an area of debate. When compared to nonopioid pain medication, opioids do not significantly improve pain intensity or pain-related function [6]. Furthermore, a recent study revealed that opioids do not provide greater pain relief at 6 weeks than placebo [7]. Considering these findings, opioid prescribers should be cautious regarding the use of opioids for pain management. The 2022 Centers for Disease Control Clinical Practice Guideline for Prescribing Opioids for Pain recommend the maximal use of nonopioid pain relievers and the prescription of opioids as needed [8]. Importantly, although these guidelines provide some recommendations regarding the process of opioid tapering, they do not describe interventions to aid physicians in this process. There now exists state-level policies, monitoring programmes, and alternative therapies to help prevent opioid over-prescription [9]. Interventions targeted at opioid prescribing behaviours of healthcare providers may be effective in reducing opioid-related harms [10–12]. Examples include prescriber education, prescription drug monitoring programmes (PDMPs), pain clinic legislation (e.g. laws requiring board-certification in pain management), and clinical guidelines [13, 14]. Considering over-prescription was the trigger of the current opioid crisis, deprescribing methods targeted at opioid prescribers are essential interventions whose effects must be analysed.

Chronic non-cancer pain (CNCP, defined as pain of non-cancer origin that lasts for 3 months or more [15]) is a common indication for opioid prescription. Over-prescription of opioids for CNCP was identified as a contributor to the opioid epidemic [16]. As a result, interventions targeting the prescription of opioids for CNCP were introduced. An overview of systematic reviews on this issue is needed to consolidate the data available due to the broad scope of available evidence. We therefore conducted an overview of systematic reviews of the effect of interventions targeting the behaviours of physician opioid prescribers for CNCP among adults on patient and population health outcomes and prescriber behaviour.

## Methods

This overview of systematic reviews was guided by Chapter V of the *Cochrane Handbook for Systematic Reviews of Interventions *[17], along with elements from additional guidance documents described in a recent review [18]. The protocol for this systematic review is registered to PROSPERO (CRD42020156815) [19].

### Search strategy

MEDLINE, Embase, and PsycInfo via Ovid; the Cochrane Database of Systematic Reviews; and Epistemonikos was systematically searched by a health sciences librarian (G.G) from inception to February 14th, 2022, and updated September 25th, 2023. Terms searched included ones related to study design such as “meta-analysis” and “health technology assessment” as well as ones related to the topic of opioids such as “analgesics, opioid” and “narcotics”. Search strategies for all databases are presented in Additional file 2: Tables S1-S5.

### Eligibility criteria

#### Population

We restricted inclusion to studies on healthcare professionals who prescribe opioids, with a focus on physician opioid prescribers. For the purposes of this overview, “physician opioid prescribers” were defined as medical doctors who prescribe opioids to patients with CNCP. Reviews limited to studies of interventions delivered exclusively to non-physician healthcare professionals (e.g. dentists, nurse practitioners, physician assistants, pharmacists) were ineligible, as were reviews limited to studies of interventions delivered exclusively or in part to patients (e.g. structured pain management programmes). Inclusion was restricted to systematic reviews, with or without meta-analysis where the systematic review methods were described in detail and a formal risk of bias assessment was performed.

#### Intervention

Any type of intervention aimed at impacting opioid prescribing behaviour for adult CNCP in an outpatient setting was included. Reviews limited to studies of interventions exclusively targeting paediatric prescription, non-CNCP prescription, or palliative pain management were excluded, as were reviews limited to studies exclusively targeting prescribing in an inpatient setting.

#### Comparators

We included systematic reviews with comparative or non-comparative studies. Comparative studies were those that compared the intervention of interest against no intervention, usual care procedures, or another active comparator. Non-comparative studies simply reported outcomes from previous data.

#### Outcomes

Eligible systematic reviews reported outcomes pertaining to the effect of the intervention of interest on patient and population health outcomes or opioid prescribing behaviour. Eligible patient and population health outcomes included changes in patient-reported health and pain outcomes, changes in opioid-related morbidity and mortality, and changes in prevalence or incidence of self-reported non-medical prescription opioid use or recreational opioid use. Eligible opioid prescribing behaviour outcomes included reduction in opioid prescribing or dose, changes in rates of prescribing of and referrals to non-pharmacological pain management therapies, and changes in intervention adherence.

### Study selection

Search results from each database were downloaded into EndNote and subsequently imported into DistillerSR [20] (Evidence Partners, Ottawa, Canada), where duplicates were identified and removed. Records were screened using a three-stage process. Two reviewers (K.P. and L.H for initial search and K.P. and A.M. for updated search) first independently screened the titles of identified citations for eligibility, then abstracts, and finally full texts. Disagreements were resolved by consensus.

### Data extraction

Two independent reviewers (K.P. and L.H.) extracted data on systematic review characteristics, interventions, outcomes, and risk of bias. Disagreements were resolved by consensus. We extracted data into five tables and assessed the overlap between primary studies and interventions using citation matrices. The citation matrix evaluates the risk of bias associated with the inclusion of systematic reviews containing the same primary studies. Our citation matrices were used to calculate corrected covered area (CCA) scores by intervention type using the following formula: [21]

$$CCA= \frac{N-r}{\left(r \times c\right)-r},$$

Where *N* is the total number of primary studies across all reviews (including duplicates), *r* is the number of unique primary studies across all reviews, and *c* is the number of reviews. We did not exclude any systematic reviews due to overlap.

### Risk of bias

The risk of bias of included systematic reviews was assessed using the Risk of Bias in Systematic Reviews (ROBIS) tool and the AMSTAR-2 tool (A MeaSurement Tool to Assess systematic Reviews-2) [22]. ROBIS assesses bias concerns in four domains: study eligibility criteria, identification and selection of studies, data collection and study appraisal, and synthesis and findings. AMSTAR-2 evaluates both systematic reviews containing randomized controlled trials (RCT) and non-RCT studies and evaluates similar domains as ROBIS. We did not exclude any systematic reviews based on risk of bias results.

## Results

Our search yielded 3420 potentially eligible citations (Additional file 1: Fig. 1). After the removal of duplicates and application of our inclusion criteria, three systematic reviews that examined 68 unique primary studies were included [23–25]. These reviews were published in 2020 and 2021 with primary studies being published from 2005 to 2021. We included two types of interventions in our overview of reviews: prescriber education and PDMPs. The overlap for prescriber education was 0% since there was only one systematic review evaluating this intervention and no overlap between primary studies included in this review. The overlap for PDMPs was 3% since only two out of 64 primary studies were duplicates (Additional file 2: Table S6).

### Characteristics of included systematic reviews

The three included systematic reviews were written by Mathieson et al., Picco et al., and Puac-Polanco et al. [23–25]. Mathieson et al. evaluated prescriber education interventions and contained two eligible primary studies (both RCTs), while Picco et al. and Puac-Polanco et al. evaluated PDMPs and contained 41 (20 cross-sectional surveys, 11 qualitative interviews, 2 mixed methods studies, 3 qualitative focus groups, 2 pre-post studies, 3 prospective studies, 1 quasi-experimental study) primary studies, and 27 (PDMP assessments) primary studies, respectively (Additional file 1: Table 1). The two systematic reviews evaluating PDMPs were conducted in the USA while the systematic review that evaluated prescriber education programmes did not report its country of origin. The two systematic reviews evaluating PDMPs conducted meta-analyses while the one evaluating prescriber education did not. Out of the total number of included primary studies, 68 evaluated PDMPs (97%) and two evaluated prescriber education (3%).

### Risk of bias assessment of included systematic reviews

Using ROBIS, two systematic reviews were rated at high risk of bias and one at low risk of bias (Additional file 1: Table 2). Among the four domains evaluated, all three systematic reviews had low risk of bias for study eligibility criteria and identification and selection of studies. For data collection and study appraisal, Picco et al. had a high risk of bias because of uncertainty regarding the data extraction procedure. No information on sensitivity analysis or study heterogeneity was explored in Puac-Polanco et al.’s systematic review. Biases in primary studies were not addressed by Mathieson et al., and they addressed this absence in the “Discussion” of the article. For this reason, the article has a low risk of bias. According to AMSTAR-2, the review with the highest risk of bias was Picco et al., followed by Puac-Polanco et al., and then Mathieson et al. (Additional file 1: Table 3). Picco et al. and Puac-Polanco et al. did not discuss heterogeneity or high risk of bias in their primary studies when interpreting their results. All three reviews were incomplete in their explanation of review methods. Both tools reveal that all included studies present moderate to high risk of bias and, therefore, limit the confidence of our analysis of their results.

### Risk of bias assessments of primary studies

The risk of bias tools used in the systematic reviews to assess the primary studies included were the Cochrane Risk of Bias version 1 (Mathieson et al.), the Mixed Methods Appraisal Tool (MMAT; Picco et al.), and the Newcastle–Ottawa Scale for Cohort Studies (Puac-Polanco et al.) [26–28]. Out of 68 primary studies, 18 were deemed at high risk of bias and 23 at fair risk of bias (Additional file 2: Tables S7-S9).

### Types of interventions

The included systematic reviews evaluated prescriber education and PDMPs. Mathieson et al. analysed prescriber education, and Picco et al. and Puac-Polanco et al. analysed PDMPs [23–25]. The outcomes reported by all three systematic reviews were split into two categories: prescriber behaviour outcomes and patient health-related outcomes. Examples of each can be found in Table 4 (Additional file 1: Table 4). It is important to note that all three systematic reviews rarely evaluate the same outcomes. The only overlapping occurs between Mathieson et al. and Puac-Polanco et al. since they both reported data for the “reduction of opioid prescriptions/use” outcome. All other outcomes were only analysed by one systematic review, affecting our ability to synthesize the information presented in these reviews.

#### Prescriber education and its effects on prescriber behaviour and opioid-related health outcomes

##### Mathieson et al. [25]

This systematic review evaluated various educational interventions for prescribers caring for patients with CNCP. The educational interventions included in this systematic review aimed to guide physicians towards appropriate prescribing behaviours through activities such as workshops, seminars and feedback sessions. We eliminated ten primary studies that evaluated patient-focused interventions [29–37]. The two remaining studies included were those by Liebschlutz et al. and Trudeau et al. [38, 39]. Liebschlutz et al. investigated the prescriber education intervention called TOPCARE, while Trudeau et al. investigated an online learning platform for prescriber education. No quantitative data were reported from the Trudeau et al. study; however, they did report observing a decrease in prescribing post online learning intervention [39]. The TOPCARE intervention is a multicomponent primary care-based programme evaluated in 53 clinicians and 985 CNCP patients [40]. This intervention had multiple steps over 12 months where patients’ pain histories were recorded and prescribers were oriented towards electronic decision tools and participated in academic detailing sessions. Liebschlutz et al. observed a 47.1% reduction of prescriptions in the intervention group vs 35.8% in the control group (risk difference − 0.1, 95% CI − 0.2, to − 0.1]), suggesting the prescriber education intervention was effective (Additional file 1: Table 5) [38]. They also reported a favourable long-term reduction in prescribed daily opioid dose among CNCP patients post-intervention (risk difference − 0.1, 95% CI − 0.2 to − 0.0). The number of discontinued opioid prescriptions in the intervention group was 21.3% versus 16.8% in the control group (risk difference − 0.1, 95% CI − 0.1 to 0.0]). Mathieson et al. reports Liebschutz et al.’s findings of a significant reduction in daily opioid dose in the intervention group vs the control group, measured using mean difference in milligram morphine equivalents (− 5.3 MME/day, 95% CI − 6.2 to − 4.5). However, one study is not sufficient to draw conclusions on the impact of this intervention on physician’s opioid prescribing behaviours. More research is needed to support prescriber education as a valuable intervention.

#### PDMPs and their effects on prescriber behaviour and opioid-related health outcomes

##### Picco et al. [23]

This systematic review evaluated several opioid prescribing outcomes, notably change in opioid prescribing habits, prescription of alternate medications, initiation of risk mitigation strategies, communication frequency between healthcare providers and patients, refusal to treat, and referral to specialized services. Here we report our observations regarding the effect of PDMPs on each of these outcomes.

Twelve primary studies evaluated opioid prescription reduction post PDMP [41–52]. PDMPs are state-wide programmes that aim to provide healthcare providers and law enforcement with prescription history and patient information in an effort to control the dispensing of drugs and occurrence of addiction [53]. A population of 2877 prescribers was included in these studies. Most prescribers (53%) reduced their opioid prescribing after using the PDMP [23]. No specifications regarding the nature of the reduction (dose or frequency) were provided in the review. The opposite was also observed in eight out of 12 primary studies that reported on increases in opioid prescribing post PDMP [42, 43, 45, 47–49, 54–56]. These primary studies had a total population size of 840 prescribers and reported that 37% of the prescribers surveyed increased their opioid prescriptions post PDMP. The change was described as an increase in opioid dose or overall quantity post PDMP. In a population of 2605 prescribers across six primary studies evaluating the use of alternate medications, 37% began prescribing alternate medications after PDMP implementation [42, 45, 52, 57–59]. Risk mitigation strategies, such as initiating or reviewing medication contract agreements, and conducting drug screening tests were undertaken post PDMP implementation. No quantitative data were reported to support this finding. In 18 primary studies, PDMPs facilitated communication between prescribers and pharmacists. Discussion between patients and providers increased post PDMP, with more physicians discussing with their patients about possible substance use disorder. Picco et al. did not provide any concrete quantitative data to support these claims. In addition, more prescribers referred patients to additional services such as psychiatric clinics and pain management clinics when information in the patient’s PDMP file suggested a potential for misuse of opioids [23]. Mixed results regarding stigmatizing behaviours from providers towards patients were observed post PDMP implementation (Additional file 1: Table 6). Stigmatizing behaviours was classified as providers describing their patients as “problem patients” or “bad patients” and describing using PDMPs to “weed out” or “purge” their practices of difficult patients.

##### Puac-Polanco et al. [24]

Like Picco et al., Puac-Polanco et al. reported a reduction in opioid prescribing behaviours post PDMP [23, 24]. This included a decrease in prescribed milligram morphine equivalents (MME) of opioids, a decrease in total volumes of opioids, a decrease in the number of overlapping prescriptions, a decrease in the number of opioid prescriptions from high-risk prescribers, and a reduction in opioid use claims among Medicaid beneficiaries. No quantitative data is available to support the claim that PDMPs decreased MME of opioids. Overall, 68% of the primary studies examining prescriber behaviour changes reported a reduction in opioid prescribing behaviours.

This systematic review also reported outcomes related to specific PDMP characteristics, mainly mandated programme use. There exists some evidence to suggest that, compared to non-mandated use, obligatory PDMP use could help reduce opioid prescription, hospitalization and opioid-related deaths. However, more research involving the specific features of PDMPs is required to understand which components help alter prescriber behaviour. Other features included monitoring more than schedule II drugs and required PDMP registration. No data regarding health outcomes was reported for these features.

#### Impact of PDMPs on patient and population health outcomes

Post PDMP implementation, rates of opioid-related hospital admissions, inpatient rehabilitation visits, and substance use disorder rates were reduced. The number of emergency department visits related to opioids as well as opioid-related poisonings was reduced with PDMPs. Mixed results were observed for opioid-related deaths, with half of the eight primary studies that evaluate this outcome confirming a reduction in opioid-related deaths [60–62] while the other half reported an increase [63] or no change [56, 63, 64]. In all, 87% of the primary studies examining opioid-related deaths or substance abuse/misuse reported a reduction in these outcomes. The population size affected by this conclusion is unknown.

## Discussion

This overview of systematic reviews was designed to synthesize the systematic review evidence on the effect of interventions targeting the behaviours of physician opioid prescribers for CNCP in adults on patient and population health outcomes and prescriber behaviour. With only three included systematic reviews, we found that there is very little systematic review evidence available that examines the effects of prescriber focused interventions on opioid prescribing behaviour in CNCP patients. One of the included systematic reviews only contained two eligible primary RCT studies, which causes potential bias. Furthermore, the primary studies in this area did not evaluate the same outcomes, making synthesis difficult. In addition, outcomes measured were sometimes vague (e.g. measuring the impact of PDMPs on “clinical decision making”), making it difficult to compare some outcomes across studies. Moreover, the systematic reviews rarely overlapped with regard to the outcomes they evaluated. We also observe a minimal overlap between PDMP primary studies (3%). This could be due to the difference in how each study measured their outcomes. Picco et al. included studies that reported on healthcare providers’ self-reported changes in clinical decision making while Puac-Polanco et al. have included observational studies that measure changes in opioid prescribing behaviours using health administrative databases. Thus, there is little overlap due to the difference in outcome measures and data sources. The scientific community requires more systematic reviews with overlapping outcomes, with quantitative analysis and that evaluate a wider range of prescriber interventions to better understand the effects of these interventions on opioid prescribing behaviours and patient outcomes. Outcome evaluation is central to the internal validity of a study, and it is essential that these outcomes are assessed using a transparent, reproducible, and objective approach. Additionally, a high proportion of primary research, especially involving pain, is often of poor methodological quality or high risk of bias [56]. The systematic reviews included in this overview draw conclusions based off such studies, as is apparent in their risk of bias assessments. Since these studies are the foundation of this overview, we remained critical in our analyses of its results and our subsequent recommendations. There remains a need for high-quality primary studies in this area with well-defined outcomes that evaluate a wider range of prescriber interventions to better understand the effects of these interventions on opioid prescribing behaviours and patient outcomes.

PDMPs have already been widely adopted across the USA [13]. Currently, all US states other than Missouri have implemented PDMPs in clinical practice as they are considered the most promising state-level intervention for controlling the prescription of opioids, informing clinicians, and protecting patients at risk of overdose [65, 66]. However, the purpose of the PDMP does not include monitoring prescribing practices in physicians. Rather, its purpose is to ensure that opioids are not being misused by patients [65]. The underlying purpose of PDMPs may explain in part the observed mixed results when used in clinic [67]. For example, the state of Kentucky has been at the forefront of PDMP use in America; however, it continues to experience the adverse effects of the opioid epidemic, underscoring the need to re-evaluate the programme or its implication [68].

Systematic reviews and overviews of systematic reviews are commonly used to help guide clinical guidelines. However, as our study demonstrates, there are not many syntheses of primary research for opioid prescriber behaviour and population health outcomes in CNCP patients available that can reliably inform these guidelines. Additional research on prescriber interventions and subsequent knowledge syntheses can help inform future clinical guidelines in this area. In 2016, the Centers for Disease Control released guidelines on the prescription of opioids for CNCP [13]. These guidelines suggested stringent restrictions on dose and duration of opioid prescriptions, as well as when and how these medications should be prescribed [13]. These restrictions were subsequently shown to have resulted in important harms, with many patients suffering severe withdrawal symptoms, undertreated pain, and psychological distress [8, 69]. Many of these restrictions we removed from the 2022 update [8]. The revised guidelines emphasize general principals and caution physicians as to the risks of specific opioid prescribing behaviours instead of explicitly advising against their use in an effort to provide individualized equitable pain management to patients [8]. The fluctuations in recommendations demonstrate the complexity of this issue as well as the need for further research on benefits and risks of using opioids for pain management. Furthermore, the long-term effects of prescriber education and PDMPs on opioid prescribing behaviours and patient health outcomes remain unknown. Previous reports classifying interventions by effectiveness related to patient safety have deemed education as low in this hierarchy [70]. It is useful to know whether this holds true for educational interventions targeted at opioid prescribers. Our overview of systematic reviews identified only two systematic reviews evaluating the effects of PDMPs on opioid prescribing behaviours and patient health outcomes in CNCP patients, highlighting an important knowledge gap regarding this specific patient population. There is currently insufficient evidence available to effectively use PDMPs to inform policy and guide clinical practice. Further studies of the effect of this nation-wide programme are needed as it has the potential to help policy makers mitigate the opioid epidemic.

Our overview has potential limitations. First, the evidence considered in this overview was restricted to that available from systematic reviews in this area. Consequently, some interventions and outcomes assessed in the primary literature were not included in the present study. Second, this overview may have been affected by the limitations of the primary studies and systematic reviews (e.g. language bias, publication bias) that were included. Third, our overview of reviews included few studies of prescriber education interventions; in total, we were only able to include one systematic review and two primary studies that discussed this topic. Lastly, an important limitation and source of heterogeneity in the form of differing definitions and components of each PDMP must also be considered. Certain constituents of a PDMP, such as mandated use or registration, could have significant effects on prescriber behaviour and patient health outcomes. However, no standardized method to evaluate this variable data exists to date, affecting the generalizability of results. Further research is required to identify such a method.

## Conclusions

Our study was designed to synthesize the systematic review evidence on the effect of interventions targeting the behaviours of physician opioid prescribers for CNCP in adults on patient and population health outcomes and prescriber behaviour. We found that there is very little evidence available in the literature that examines the effects of prescriber focused interventions on opioid prescribing behaviour in CNCP patients [23–25]. The information we synthesized from the three included systematic reviews does not definitively demonstrate that any of the assessed interventions are beneficial due to high risk of bias and mixed results. Importantly, the studies included all contain significant risk of bias, thus requiring their results to be interpreted with caution. There is some question about whether PDMPs are beneficial but, given the magnitude of the opioid crisis, high-quality primary studies and systematic reviews are needed to help guide healthcare workers managing patients on opioids.

### Supplementary Information

**Additional file 1: Tables and figures. Table 1. **Characteristics of included systematic reviews of interventions targeting prescribing behaviour of opioids for chronic non-cancer pain.** Table 2. **Risk of Bias Assessment for Systematic Reviews Using the ROBIS Tool. **Table 3. **Risk of Bias Assessment for Systematic Reviews Using the AMSTAR-2.** Table 4. **Characteristics of prescriber education and PDMPs interventions for physician opioid prescribers evaluated by included systematic reviews.** Table 5. **Summary of findings reported by included systematic reviews (n = 3) on the impact of interventions on prescriber behaviour and patient and population health outcomes. **Table 6. **Results and conclusions of each included systematic review on prescriber interventions for CNCP. **Figure 1. **PRISMA flow diagram for systematic reviews assessing physician-targeted interventions for chronic non-cancer pain.

**Additional file 2: Supplementary. Table S1. **Search Strategy to Identify Eligible Systematic Reviews (MEDLINE via Ovid).** Table S2. **Search Strategy to Identify Eligible Systematic Reviews (Embase via Ovid).** Table S3. **Search Strategy to Identify Eligible Systematic Reviews (PsycINFO via Ovid).** Table S4. **Search Strategy to Identify Eligible Systematic Reviews (Cochrane Database of Systematic Reviews).** Table S5. **Search Strategy to Identify Eligible Systematic Reviews (Epistemonikos).** Table S6. **Citation Matrix.** Table S7. **Risk of Bias of Primary Studies for Mathieson et al. using the Cochrane V1 Risk of Bias Assessment.** Table S8. **Risk of Bias of Primary Studies for Puac-Polanco et al. using the Ottawa Newcastle Assessment Method. **Table S9. **Risk of Bias of Primary Studies for Picco et al. using the Mixed Methods Appraisal Tool

## Data Availability

Not applicable.

## Abbreviations

AMSTAR-2: A MeaSurement Tool to Assess systematic Reviews—2
CCA: Corrected covered area
CNCP: Chronic non-cancer pain
MMAT: Mixed methods appraisal tool
MME: Milligram morphine equivalent
PDMP: Prescription drug monitoring programme
RCT: Randomized controlled trial
ROBIS: Risk of bias in systematic review
