# Supplementary material for: Efficacy of interventions targeted at physician prescribers of opioids for chronic non-cancer pain: an overview of systematic reviews
Source: BMC Med. 2024 Feb 20;22:76. doi: 10.1186/s12916-024-03287-1 (PMC10877926; doi:10.1186/s12916-024-03287-1)
Supplement: Supplementary file 1 — Additional file 1: Tables and figures. Table 1. Characteristics of included systematic reviews of interventions targeting prescribing behaviour of opioids for chronic non-cancer pain. Table 2. Risk of Bias Assessment for Systematic Reviews Using the ROBIS Tool. Table 3. Risk of Bias Assessment for Systematic Reviews Using the AMSTAR-2. Table 4. Characteristics of prescriber education and PDMPs interventions for physician opioid prescribers evaluated by included systematic reviews. Table 5. Summary of findings reported by included systematic reviews (n = 3) on the impact of interventions on prescriber behaviour and patient and population health outcomes. Table 6. Results and conclusions of each included systematic review on prescriber interventions for CNCP. Figure 1. PRISMA flow diagram for systematic reviews assessing physician-targeted interventions for chronic non-cancer pain. [file 12916_2024_3287_MOESM1_ESM.docx]

**Table 1.** Characteristics of included systematic reviews of interventions targeting prescribing behaviour of opioids for chronic non-cancer pain

| **Study Author, Year** | **Databases Searched, Search Period** | **Objectives** | **Population** | **Interventions** | **Comparators** | **Outcomes examined** | **Study Design** | **Primary Studies and Quality Assessment** | **Funding Source** |
| --- | --- | --- | --- | --- | --- | --- | --- | --- | --- |
| **Prescriber Education** | | | | | | | | | |
| **Mathieson 2020** | Databases: Pub- Med (Legacy), MEDLINE, EMBASE, PsycINFO, Web of Science (Core Collection), Cochrane Central Register of Controlled Trials, International Pharmaceutical Abstracts, ClinicalTrials.gov, World Health Organization International Clinical Trials Registry Platform Search period: Inception to 13th January 2020 | Evaluation of interventions to deprescribe opioid medication | Healthcare providers whose patients are adults (≥ 18 years) with chronic pain | TOPCARE & online education for prescribers | Interventions in patients with chronic pain versus control. | Mean reduction of daily dose of opioids, reduction of prescriptions, cessation/reduction of opioid use, adverse events, change in pain intensity, disability and quality-of-life scores. | Randomized controlled trials (RCTs) | Total: 2 (2017)*  RCTs Quality Assessment: Cochrane Risk of Bias (V1) | Sydney Medical School, The University of Sydney, Public Health Research Collaboration Scheme |
| **PDMPs** | | | | | | | | | |
| **Picco 2021** | Databases: MEDLINE, Embase, PubMed, PsycInfo, CINAHL, Web of Science, Scopus  Search period: Inception - April 2021 | To identify the ways in which PDMP implementation affect clinical decision making | All healthcare providers who prescribe controlled substances and have access to PDMPs (physicians, nurse practitioners, dentists, osteopaths and pharmacists). | Prescription Drug Monitoring Programs (PDMPs) | Pre PDMP vs post PDMP | Influence of PDMPs on healthcare providers’ clinical decision making (prescription behaviour, withholding of treatment etc.) | Qualitative interviews, Prospective quasi- experimental, Cross sectional survey, Qualitative focus groups | Total : 41 (2005-2021) 22 Cross sectional surveys 8 Qualitative interviews 2 Mixed-methods studies 2 Qualitative focus groups  2 Pre-post studies 2 Prospective studies  1 Quasi-experimental study Quality Assessment: MMAT |  |
| **Puac-Polanco 2020** | Databases: Embase, Google Scholar, MEDLINE, Epub Ahead of Print, In-Process & Other Non-Indexed Citations, Daily and Versions, PubMed, Web of Science Core Collection Search period: Any study - January 9, 2019 | To investigate prescription opioid related outcomes after PDMP implementation | Patients affected by PDMP | Prescription Drug Monitoring Programs (PDMPs) | Pre PDMP vs post PDMP | Opioid prescribing behaviours, opioid supply or diversion, opioid-related morbidity and substance-use disorders, addressed opioid-related deaths | Included studies assessed at least 1 of the 4 prescription opioid–related outcomes, reported quantitative data or measured impact of PDMP vs no PDMP and were US studies in English. Observational research designs like before and after comparison time-series analysis, or prospective and retrospective cohorts | Total: 29 (2009-2019)  Observational studies Quality Assessment: Ottawa Newcastle | National Center for Injury Prevention and Control, the Centers for Disease Control and Prevention (grant R49CE002096) and the National Institutes of Health (grants R25GM062454 to V.P.-P; |

****This systematic review had 12 primary studies, however 10 were ineligible for our overview because they focused on patient interventions instead of prescriber interventions per our eligibility criteria***

**Table 2:** Risk of Bias Assessment for Systematic Reviews Using the ROBIS Tool

|  | **Study Eligibility Criteria** | **Identification and Selection of Studies** | **Data Collection and Study Appraisal** | **Synthesis and Findings** | **Review** |
| --- | --- | --- | --- | --- | --- |
| **Mathieson et al.** | Low | Low | Low | High | Low |
| **Picco et al.** | Low | Low | High | High | High |
| **Puac-Polanco et al.** | Low | Low | Low | Unclear | High |

|  | Mathieson 2020 | Picco 2021 | Puac-Polanco 2020 |
| --- | --- | --- | --- |
| Did the research questions and inclusion criteria for the review include the components of PICO? | Yes | Yes | Yes |
| Did the report of the review contain an explicit statement that the review methods were established prior to the conduct of the review and did the report justify any significant deviations from the protocol? | No | No | No |
| Did the review authors explain their selection of the study designs for inclusion in the review? | No | Yes | No |
| Did the review authors use a comprehensive literature search strategy? | Yes | No | Partial Yes |
| Did the review authors perform study selection in duplicate? | Yes | Yes | Yes |
| Did the review authors perform data extraction in duplicate? | Yes | No | Yes |
| Did the review authors provide a list of excluded studies and justify the exclusions? | Partial Yes | Yes | Yes |
| Did the review authors describe the included studies in adequate detail? | Yes | Partial Yes | Partial Yes |
| Did the review authors use a satisfactory technique for assessing the risk of bias (RoB) in individual studies that were included in the review? | Yes | Yes | Yes |
| Did the review authors report on the sources of funding for the studies included in the review? | No | No | No |
| If meta-analysis was performed did the review authors use appropriate methods for statistical combination of results? | No meta-analysis | Yes | No meta-analysis |
| If meta-analysis was performed, did the review authors assess the potential impact of RoB in individual studies on the results of the meta-analysis or other evidence synthesis? | No meta-analysis | No | No meta-analysis |
| Did the review authors account for RoB in individual studies when interpreting/discussing the results of the review? | Yes | No | No |
| Did the review authors provide a satisfactory explanation for, and discussion of, any heterogeneity observed in the results of the review? | Yes | No | No |
| If they performed quantitative synthesis did the review authors carry out an adequate investigation of publication bias (small study bias) and discuss its likely impact on the results of the review? | No meta-analysis | No | No meta-analysis |
| Did the review authors report any potential sources of conflict of interest, including any funding they received for conducting the review? | Yes | Yes | Yes |

**Table 3:** Risk of Bias Assessment for Systematic Reviews Using the AMSTAR-2

**Table 4.** Characteristics of prescriber education and PDMPs interventions for physician opioid prescribers evaluated by included systematic reviews.

| Systematic Review | Program Details | Target Population | Target Prescription Indications | Target Prescription Settings | Major Components | Objectives | Country/ Jurisdiction |
| --- | --- | --- | --- | --- | --- | --- | --- |
| Prescriber Education | | | | | | | |
|  |  |  |  |  |  |  |  |
| Mathieson 2020 | TOPCARE for physicians (Liebschlutz et al.) | Primary Care Clinicians | Physicians of patients with chronic pain receiving long term opioid therapy | Primary care clinics | TOPCARE included 4 components such as pain history taking by a nurse, academic detailing sessions, education about electronic decision tools and population management. Intervention lasted for 12 months. | To determine whether a multicomponent intervention improves guideline adherence while decreasing opioid misuse risk. | USA |
|  | Online education for physicians (Trudeau et al) | Primary Care Clinicians | Physicians of patients with CNCP | Primary care clinics | Online education through the (Managing Addiction and Pain in Primary Care (MAP-PC) programme, focusing on the management of chronic pain and addiction to reduce prescribing behaviour. | To improve pain management practices, we developed an online interactive continuing education (CE) program for primary care providers (PCPs). | USA |
| PDMPs | | | | | | | |
|  |  |  |  |  |  |  |  |
| Picco  2021 | State-wide PDMP implementation | Healthcare providers | Opioid prescribers from a variety of disciplines | Private practice, hospitals, pharmacies | N/A | To determine if PDMPs have an impact on clinical decision making and opioid prescribing behaviour | USA |
| Puac-Polanco 2020 |  |  |  |  |  |  | USA |


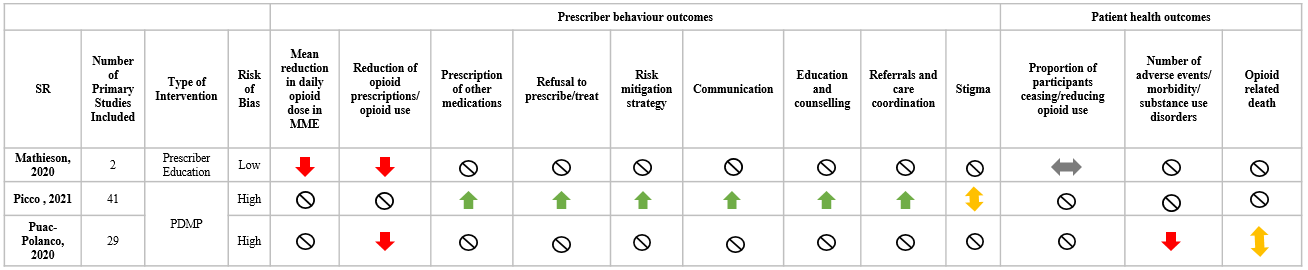
**Table 5.** Summary of findings reported by included systematic reviews (n=3) on the impact of interventions on prescriber behaviour and patient and population health outcomes.

Decrease in the outcome; increase in the outcome; mixed effect; no effect was observed; outcome was not evaluated

**Note**: It is important to note that in their systematic review, Mathieson et al. included primary studies that also analysed patient-focused interventions^29-37,83^. Given the eligibility criteria of this overview, we restricted inclusion to their findings regarding the prescriber-focused interventions. Mathieson et al. included two primary studies that evaluated interventions aimed at changing prescriber behaviour (Trudeau et al. and Liebschultz et al.)^38,39^. However, they only report data from Liebschultz et al^38^. Therefore, our assessment of interventions aimed at prescriber behaviour only includes data from one systematic review containing one primary study for this type of intervention.

**Table 6.**  Results and conclusions of each included systematic review on prescriber interventions for CNCP.

| Author | Target Population | Interventions Examined | Outcomes | Interpretation of Results | Summary/Conclusions from SRs |
| --- | --- | --- | --- | --- | --- |
| Prescriber Education | | | | | |
| Mathieson  2020 | Opioid prescribers of patients ≥18 yrs of age with CNCP in a clinical setting n = 1126 | Prescriber education | Mean reduction of daily dose of opioids (in MME)  Reduction of opioid analgesic prescriptions Opioid cessation rates  Adverse events  Mean change in pain intensity, disability and quality-of-life scores. | One clinician-focused intervention reported reduction in the number of opioid prescriptions given post intervention.  No change is cessation or discontinuation rate amongst patients.  Other outcomes were only examined for patient focused outcomes. | Possibility that clinician focused interventions work in the long-term. |
| PDMPs | | | | | |
| Picco 2021 | Healthcare prescribers responsible for prescribing opioids.  n = 11,369 | Prescription Drug Monitoring Programs (PDMPs) | The supply of controlled substances  Refusal to prescribe or treat Risk mitigation strategies Communication  Education and counselling  Referrals and care coordination  Stigma | Supply of controlled substances: 53 % decreased prescribing prevalence 19% increased prescribing prevalence 37% Prevalence of prescription of alternative medication Increases in refusal to prescribe or treat, use of risk mitigation strategies, communication, patient education and referrals to specialty clinics.  Mixed effects on stigma related to opioid use. | PDMP use positively influenced healthcare providers’ clinical decision-making. |
| Puac-Polanco 2020 | Eligible prescribers not specified  n = population affected by the PDMPs. | Prescription Drug Monitoring Programs (PDMPs) | Opioid prescribing behaviours  Opioid related morbidity or substance use disorder Addressed opioid related deaths | 11/16 studies reported reduction in opioid prescription post PDMP.  7/8 studies reported changes in opioid related morbidity.  Mixed effects for opioid related deaths (half report reduction, half report increase or no effect). | PDMPs incited reductions in opioid prescribing, opioid-related morbidity and substance-use disorders |

**Figure 1.** PRISMA flow diagram for systematic reviews assessing physician-targeted interventions for chronic non-cancer pain.

Ovid

EMBASE

(n=2,731)

Ovid Medline

(n=1116)

Epistemo-nikos

(n=443)

Ovid

PsycINFO

(n=242)

Cochrane

Reviews

(n=111)

Total records identified from search

(n=4643)

Other sources

(n=1)

Duplicates removed

(n=1226)

Potentially relevant articles screened by title and abstract

(n=3417)

Records excluded

(n=3355)

Full-text articles assessed for eligibility

(n=62)

Full-text articles excluded (n=59)

- Ineligible population (n= 32)
- Ineligible outcomes (n = 9)
- Ineligible form of publication (n = 3)
- Ineligible study design (n = 2)
- Unsuitable ROB (n = 3)
- Missed duplicate (n = 1)
- Abstract (n = 5)

Eligible studies (all opioids interventions)

(n=3)
