# Supplementary material for: Efficacy of interventions targeted at physician prescribers of opioids for chronic non-cancer pain: an overview of systematic reviews
Source: BMC Med. 2024 Feb 20;22:76. doi: 10.1186/s12916-024-03287-1 (PMC10877926; doi:10.1186/s12916-024-03287-1)
Supplement: Supplementary file 2 — Additional file 2: Supplementary. Table S1. Search Strategy to Identify Eligible Systematic Reviews (MEDLINE via Ovid). Table S2. Search Strategy to Identify Eligible Systematic Reviews (Embase via Ovid). Table S3. Search Strategy to Identify Eligible Systematic Reviews (PsycINFO via Ovid). Table S4. Search Strategy to Identify Eligible Systematic Reviews (Cochrane Database of Systematic Reviews). Table S5. Search Strategy to Identify Eligible Systematic Reviews (Epistemonikos). Table S6. Citation Matrix. Table S7. Risk of Bias of Primary Studies for Mathieson et al. using the Cochrane V1 Risk of Bias Assessment. Table S8. Risk of Bias of Primary Studies for Puac-Polanco et al. using the Ottawa Newcastle Assessment Method. Table S9. Risk of Bias of Primary Studies for Picco et al. using the Mixed Methods Appraisal Tool [file 12916_2024_3287_MOESM2_ESM.docx]

**APPENDIX**

**Table S1: Search Strategy to Identify Eligible Systematic Reviews (MEDLINE via Ovid)**

| **Search Number** | **Description** |
| --- | --- |
| 1 | narcotic-related disorders/ or substance-related disorders/ or exp analgesics, opioid/ or exp opioid-related disorders/ or (narcotic* or opiate* or opioid* or acetylmethadol or alfentanil or anileridine or Belladonna or Benzomorphan* or bezitramide or buprenorphine or butorphanol or Codeine or Dextromethorphan or Dextromoramide or Dextropropoxyphene or dezocine or Diamorphine or dihydrocodeine or Diphenylpropylamine or Ethylmorphine or Fentanyl* or Heroin or Hydrocodon* or Hydromorphon* or ketobemidone or levacetylmethadol or Meperidine or Meptazinol or methadone or Morphan* or Morphine* or nalbuphine or nicomorphine or normethadone or Opium or Oripavine or Oxycodone or Oxymorphone or Papaveretum or Pentazocine or pethidin* or Phenazocine or Phenoperidine or phentanyl or Phenylpiperidine or Piritramide or remifentanil or Sufentanil or sulfentanil or sulfentanyl or tapentadol or Tilidine or Tramadol*).mp. or analgesic*.ti. |
| 2 | practice patterns, physicians'/ or exp prescriptions/ or exp prescription drug monitoring programs/ or "knowledge, attitudes, practice"/ or (doctor* or physician* or practitioner* or provider* or surgeon* or dispens* or prescribe* or prescribing or deprescrib* or overprescri* or overtreat* or underprescri* or undertreat* or prescription* or script? or stewardship* or refill* or taper*).mp. |
| 3 | systematic review/ or meta analysis/ or "systematic review as topic"/ or exp "meta-analysis as topic"/ or technology assessment, biomedical/ or (meta analy* or metaanaly* or technology assessment* or hta or htas or ((evidence or mixed method* or rapid or systematic) adj3 (overview or review or metareview or metasynthesis))).ti. or (cochrane database of systematic reviews or technology assessment*).jw. |
| 4 | 1 and 2 and 3 |

Date of search: September 25^th^ , 2023

**Table S2: Search Strategy to Identify Eligible Systematic Reviews (Embase via Ovid)**

| **Search Number** | **Description** |
| --- | --- |
| 1 | exp narcotic analgesic agent/ |
| 2 | controlled substance/ |
| 3 | (narcotic* or opiate* or opioid* or acetylmethadol or alfentanil or anileridine or Belladonna or Benzomorphan* or bezitramide or buprenorphine or butorphanol or Codeine or Dextromethorphan or Dextromoramide or Dextropropoxyphene or dezocine or Diamorphine or dihydrocodeine or Diphenylpropylamine or Ethylmorphine or Fentanyl* or Heroin or Hydrocodon* or Hydromorphon* or ketobemidone or levacetylmethadol or Meperidine or Meptazinol or methadone or Morphan* or Morphine* or nalbuphine or nicomorphine or normethadone or Opium or Oripavine or Oxycodone or Oxymorphone or Papaveretum or Pentazocine or pethidin* or Phenazocine or Phenoperidine or phentanyl or Phenylpiperidine or Piritramide or remifentanil or Sufentanil or sulfentanil or sulfentanyl or tapentadol or Tilidine or Tramadol*).mp. or analgesic*.ti. |
| 4 | 1 or 2 or 3 |
| 5 | prescription/ or prescription drug monitoring program/ or (doctor* or physician* or practitioner* or provider* or surgeon* or dispens* or prescribe* or prescribing or deprescrib* or overprescri* or overtreat* or underprescri* or undertreat* or prescription* or script? or stewardship* or refill* or taper*).mp. |
| 6 | systematic review/ or exp meta analysis/ or "systematic review (topic)"/ or "meta analysis (topic)"/ or biomedical technology assessment/ |
| 7 | (meta analy* or metaanaly* or technology assessment* or hta or htas or ((evidence or mixed method* or rapid or systematic) adj3 (overview or review or metareview or metasynthesis))).ti. |
| 8 | (cochrane database of systematic review or technology assessment*).jw. |
| 9 | 6 or 7 or 8 |
| 10 | 4 and 5 and 9 |

Date of search: September 25, 2023

**Table S3: Search Strategy to Identify Eligible Systematic Reviews (PsycINFO via Ovid)**

| **Search Number** | **Description** |
| --- | --- |
| 1 | exp narcotic drugs/ or (narcotic* or opiate* or opioid* or acetylmethadol or alfentanil or anileridine or Belladonna or Benzomorphan* or bezitramide or buprenorphine or butorphanol or Codeine or Dextromethorphan or Dextromoramide or Dextropropoxyphene or dezocine or Diamorphine or dihydrocodeine or Diphenylpropylamine or Ethylmorphine or Fentanyl* or Heroin or Hydrocodon* or Hydromorphon* or ketobemidone or levacetylmethadol or Meperidine or Meptazinol or methadone or Morphan* or Morphine* or nalbuphine or nicomorphine or normethadone or Opium or Oripavine or Oxycodone or Oxymorphone or Papaveretum or Pentazocine or pethidin* or Phenazocine or Phenoperidine or phentanyl or Phenylpiperidine or Piritramide or remifentanil or Sufentanil or sulfentanil or sulfentanyl or tapentadol or Tilidine or Tramadol*).mp. |
| 2 | exp "prescribing (drugs)"/ or prescription drugs/ or (doctor* or physician* or practitioner* or provider* or surgeon* or dispens* or prescribe* or prescribing or deprescrib* or overprescri* or overtreat* or underprescri* or undertreat* or prescription* or script? or stewardship* or refill* or taper*).mp. |
| 3 | meta analysis/ |
| 4 | (systematic review or meta analysis or metasynthesis).md. |
| 5 | (meta analy* or metaanaly* or technology assessment* or hta or htas or ((evidence or mixed method* or rapid or systematic) adj3 (overview or review or metareview or metasynthesis))).ti. |
| 6 | 3 or 4 or 5 |
| 7 | 1 and 2 and 6 |

Date of search: September 25, 2023

**Table S4: Search Strategy to Identify Eligible Systematic Reviews (Cochrane Database of Systematic Reviews)**

| **Search Number** | **Description** |
| --- | --- |
| 1  Title Abstract Keyword | (narcotic* or opiate* or opioid* or acetylmethadol or alfentanil or anileridine or Belladonna or Benzomorphan* or bezitramide or buprenorphine or butorphanol or Codeine or Dextromethorphan or Dextromoramide or Dextropropoxyphene or dezocine or Diamorphine or dihydrocodeine or Diphenylpropylamine or Ethylmorphine or Fentanyl* or Heroin or Hydrocodon* or Hydromorphon* or ketobemidone or levacetylmethadol or Meperidine or Meptazinol or methadone or Morphan* or Morphine* or nalbuphine or nicomorphine or normethadone or Opium or Oripavine or Oxycodone or Oxymorphone or Papaveretum or Pentazocine or pethidin* or Phenazocine or Phenoperidine or phentanyl or Phenylpiperidine or Piritramide or remifentanil or Sufentanil or sulfentanil or sulfentanyl or tapentadol or Tilidine or Tramadol*):ti,ab,kw |
| 2  Title Abstract Keyword | (doctor* or physician* or practitioner* or provider* or surgeon* or dispens* or prescribe* or prescribing or deprescrib* or overprescri* or overtreat* or underprescri* or undertreat* or prescription* or script* or stewardship* or refill* or taper*):ti,ab,kw |
| 3 | 1 and 2 |
| Search limits | Cochrane Reviews  Cochrane Protocols |

Date of search: September 25, 2023

**Table S5: Search Strategy to Identify Eligible Systematic Reviews (Epistemonikos)**

| **Search Number** | **Description** |
| --- | --- |
| 1  (Title/ Abstract) | (Title/ Abstract) narcotic* OR opiate* OR opioid* OR acetylmethadol OR alfentanil OR anileridine OR Belladonna OR Benzomorphan* OR bezitramide OR buprenorphine OR butorphanol OR Codeine OR Dextromethorphan OR Dextromoramide OR Dextropropoxyphene OR dezocine OR Diamorphine OR dihydrocodeine OR Diphenylpropylamine OR Ethylmorphine OR Fentanyl* OR Heroin OR Hydrocodon* OR Hydromorphon* OR ketobemidone OR levacetylmethadol OR Meperidine OR Meptazinol OR methadone OR Morphan* OR Morphine* OR nalbuphine OR nicomorphine OR normethadone OR Opium OR Oripavine OR Oxycodone OR Oxymorphone OR Papaveretum OR Pentazocine OR pethidin* OR Phenazocine OR Phenoperidine OR phentanyl OR Phenylpiperidine OR Piritramide OR remifentanil OR Sufentanil OR sulfentanil OR sulfentanyl OR tapentadol OR Tilidine OR Tramadol* |
| 2  (Title/ Abstract) | (Title/ Abstract) doctor* OR physician* OR practitioner* OR provider* OR surgeon* OR dispens* OR prescribe* OR prescribing OR deprescrib* OR overprescri* OR overtreat* OR underprescri* OR undertreat* OR prescription* OR script? OR stewardship* OR refill* OR taper* |
| 3 | 1 and 2 |
| Filters | Publication type: Systematic Review  Systematic Review Question: Interventions |

Date of search: September 25, 2023

**Table S6: Citation Matrix**

| **Primary Study** | **Design** | **Country** | **Mathieson 2020** | **Puac-Polanco 2020** | **Picco 2021** |
| --- | --- | --- | --- | --- | --- |
| Liebschutz 2017 | RCT | USA | x |  |  |
| Trudeau 2017 | RCT | USA | x |  |  |
| Paulozzi 2011 | PDMP assessment | USA |  | x |  |
| Brady 2014 | PDMP assessment | USA |  | x |  |
| McAllister 2015 | pre-post survey | USA |  | x | x |
| Rasubala 2015 | PDMP assessment | USA |  | x |  |
| Rutkow 2015 | PDMP assessment | USA |  | x | x |
| Bao 2016 | PDMP assessment | USA |  | x |  |
| Chang 2016 | PDMP assessment | USA |  | x |  |
| Dowell 2016 | PDMP assessment | USA |  | x |  |
| Brown 2017 | PDMP assessment | USA |  | x |  |
| Moyo 2017 | PDMP assessment | USA |  | x |  |
| Wen 2017 | PDMP assessment | USA |  | x |  |
| Bao 2018 | PDMP assessment | USA |  | x |  |
| Chang 2018 | PDMP assessment | USA |  | x |  |
| Lin 2018 | PDMP assessment | USA |  | x |  |
| Ranapurwala 2018 | PDMP assessment | USA |  | x |  |
| Yarbrough 2018 | PDMP assessment | USA |  | x |  |
| Reisman 2009 | PDMP assessment | USA |  | x |  |
| Surratt 2014 | PDMP assessment | USA |  | x |  |
| Ali 2017 | PDMP assessment | USA |  | x |  |
| Reifler 2012 | PDMP assessment | USA |  | x |  |
| Maughan 2015 | PDMP assessment | USA |  | x |  |
| Buchmueller 2018 | PDMP assessment | USA |  | x |  |
| Pauly 2018 | PDMP assessment | USA |  | x |  |
| Grecu 2019 | PDMP assessment | USA |  | x |  |
| Patrick 2016 | PDMP assessment | USA |  | x |  |
| Nam 2017 | PDMP assessment | USA |  | x |  |
| Phillips 2017 | PDMP assessment | USA |  | x |  |
| Allen 2019 | Qualitative interviews | not stated |  |  | x |
| Baehren 2010 | Prospective quasi-experimental | not stated |  |  | x |
| Barrett and Watson 2005 | Cross sectional survey | not stated |  |  | x |
| Feldman 2011 | Cross sectional survey | not stated |  |  | x |
| Finley 2018 | Qualitative interviews | not stated |  |  | x |
| Gershman 2014 | Cross sectional survey | not stated |  |  | x |
| Goodin 2021 | Cross sectional survey | not stated |  |  | x |
| Green 2013 | Cross sectional survey | not stated |  |  | x |
| Green 2012 | Cross sectional survey | not stated |  |  | x |
| Grover and Garmel | Cross sectional survey | not stated |  |  | x |
| Hagemeier 2018 | Qualitative focus groups | not stated |  |  | x |
| Hernandez-Meier | Cross sectional survey | not stated |  |  | x |
| Hildebran 2014 | Qualitative focus groups | not stated |  |  | x |
| Hildebran 2016 | Qualitative interviews | not stated |  |  | x |
| Hussain 2019 | Qualitative interviews | not stated |  |  | x |
| Irvine 2014 | Cross sectional survey | not stated |  |  | x |
| Leichtling 2017 | Qualitative interviews | not stated |  |  | x |
| Leichtling 2019 | Mixed methods | not stated |  |  | x |
| LeMire 2012 | Cross sectional survey | not stated |  |  | x |
| Lin 2017 | Cross-sectional survey | not stated |  |  | x |
| McCauley 2019 | Cross-sectional survey | not stated |  |  | x |
| McCauley 2016 | Cross sectional survey | not stated |  |  | x |
| McDonald 2019 | Prospective controlled experiment | not stated |  |  | x |
| Norwood and Wright 2016 | Cross sectional survey | not stated |  |  | x |
| Ovadia 2020 | Cross sectional survey | not stated |  |  | x |
| Perrone 2012 | Cross sectional survey | not stated |  |  | x |
| Pett 2019 | Mixed methods | not stated |  |  | x |
| Radomski 2018 | qualitative interviews | not stated |  |  | x |
| Rickles 2021 | Cross sectional survey | not stated |  |  | x |
| Rittenhouse 2015 | Cross sectional survey | not stated |  |  | x |
| Smith 2015 | Qualitative interviews | not stated |  |  | x |
| Thomas 2014 | pre-post survey | not stated |  |  | x |
| Thornton 2020 | Qualitative focus groups | not stated |  |  | x |
| Wang 2015 | Cross sectional survey | not stated |  |  | x |
| Weiner 2013 | Prospective observational | not stated |  |  | x |
| Worley 2015 | Qualitative interviews | not stated |  |  | x |
| Young 2017 | Cross sectional survey | not stated |  |  | x |

**Table S7: Risk of Bias of Primary Studies for Mathieson et al using the Cochrane V1 Risk of Bias Assessment**

low risk of bias unclear risk of bias high risk of bias.

**Table S8: Risk of Bias of Primary Studies for Puac-Polanco et al using the Ottawa Newcastle Assessment Method**

| **Systematic Review** | **Primary Study** | **Representativeness of Exposed** | **Selection of Non-Exposed** | **Exposure Assessment** | **Outcomes Timeline** | **Between Exposed and Non-Exposed** | **Assessment of Outcome** | **Follow-Up Time** | **Follow-Up Adequacy** | **Grade** |
| --- | --- | --- | --- | --- | --- | --- | --- | --- | --- | --- |
| Puac-Polanco 2020 | Paulozzi 2011 | 1 | 1 | 1 | 0 | 1 | 1 | 1 | 1 | F |
|  | Brady 2014 | 1 | 1 | 1 | 0 | 1 | 1 | 1 | 1 | G |
|  | McAllister 2015 | 1 | 0 | 1 | 0 | 1 | 1 | 0 | 1 | F |
|  | Rasubala 2015 | 1 | 1 | 1 | 0 | 0 | 1 | 1 | 1 | P |
|  | Rutkow 2015 | 1 | 1 | 0 | 0 | 1 | 1 | 1 | 1 | F |
|  | Bao,2016 | 1 | 1 | 1 | 0 | 1 | 1 | 1 | 1 | G |
|  | Chang,2016 | 1 | 1 | 0 | 0 | 1 | 1 | 1 | 1 | F |
|  | Dowell,2016 | 1 | 1 | 0 | 0 | 1 | 1 | 1 | 1 | F |
|  | Brown,2017 | 1 | 1 | 0 | 0 | 1 | 1 | 1 | 1 | F |
|  | Moyo,2017 | 1 | 1 | 1 | 0 | 1 | 1 | 1 | 1 | G |
|  | Wen,2017 | 1 | 1 | 1 | 0 | 1 | 1 | 1 | 1 | G |
|  | Bao,2018 | 1 | 1 | 1 | 0 | 1 | 1 | 1 | 1 | G |
|  | Chang,2018 | 1 | 1 | 0 | 0 | 1 | 1 | 1 | 1 | F |
|  | Lin,2018 | 1 | 1 | 1 | 0 | 1 | 1 | 0 | 0 | P |
|  | Ranapurwala, 2018 | 1 | 1 | 1 | 0 | 2 | 1 | 1 | 1 | G |
|  | Yarbrough, 2018 | 1 | 1 | 1 | 0 | 1 | 1 | 1 | 1 | G |
|  | Reisman, 2009 | 1 | 1 | 1 | 0 | 0 | 1 | 1 | 1 | P |
|  | Surratt, 2014 | 1 | 1 | 0 | 0 | 0 | 1 | 1 | 1 | P |
|  | Ali 2017 | 1 | 1 | 1 | 0 | 1 | 1 | 1 | 1 | G |
|  | Reisman, 2009 | 1 | 1 | 1 | 0 | 0 | 1 | 1 | 1 | P |
|  | Reifler, 2012 | 1 | 1 | 0 | 0 | 0 | 1 | 1 | 1 | P |
|  | Maughan, 2015 | 1 | 1 | 1 | 0 | 1 | 1 | 1 | 1 | P |
|  | Ali, 2017 | 1 | 1 | 1 | 0 | 1 | 1 | 1 | 1 | G |
|  | Brown 2017 | 1 | 1 | 0 | 0 | 1 | 1 | 1 | 1 | F |
|  | Buchmueller, 2018 | 1 | 1 | 1 | 0 | 1 | 1 | 1 | 1 | G |
|  | Pauly, 2018 | 1 | 1 | 1 | 0 | 1 | 1 | 1 | 1 | G |
|  | Grecu 2019 | 1 | 1 | 1 | 0 | 2 | 1 | 1 | 1 | G |
|  | Paulozzi, 2011 | 1 | 1 | 1 | 0 | 1 | 1 | 1 | 1 | F |
|  | Li 2014 | 1 | 1 | 1 | 0 | 1 | 1 | 1 | 1 | G |
|  | Delcher, 2015 | 1 | 1 | 1 | 0 | 2 | 1 | 1 | 1 | G |
|  | Dowell 2016 | 1 | 1 | 0 | 0 | 1 | 1 | 1 | 1 | F |
|  | Patrick, 2016 | 1 | 1 | 1 | 0 | 2 | 1 | 1 | 1 | G |
|  | Nam, 2017 | 1 | 1 | 0 | 0 | 1 | 1 | 1 | 1 | F |
|  | Phillips, 2017 | 1 | 1 | 0 | 0 | 1 | 1 | 0 | 1 | F |
|  | Grecu, 2019 | 1 | 1 | 1 | 0 | 2 | 1 | 1 | 1 | G |

0 indicates no stars; 1 indicates one star; 2 indicates two stars. “P” indicates poor quality, “F” indicates fair quality; “G” indicates good quality.

**Table S9: Risk of Bias of Primary Studies for Picco et al using the Mixed Methods Appraisal Tool**

| **Systematic Review** | **Primary study author and year** | **MMAT Evidence Level^[[1]](#footnote-1)^** |
| --- | --- | --- |
| Picco 2021 | Allen 2019 | 100 |
|  | Beahren 2010 | 20 |
|  | Barret 2005 | 80 |
|  | Feldman 2011 | 80 |
|  | Finley 2018 | 100 |
|  | Gershman 2014 | 60 |
|  | Goodin 2021 | 60 |
|  | Green 2013 | 60 |
|  | Green 2012 | 60 |
|  | Grover 2012 | 80 |
|  | Hagemeier 2018 | 100 |
|  | Hernandez-Meier 2017 | 40 |
|  | Hidebran 2014 | 100 |
|  | Hildebran 2016 | 100 |
|  | Hussain 2019 | 100 |
|  | Irvine 2014 | 100 |
|  | Leichtling 2017 | 100 |
|  | Leichtling 2019 | 40 |
|  | LeMire 2012 | 40 |
|  | Lin 2017 | 80 |
|  | McAllister 2015 | 80 |
|  | McCauley 2019 | 100 |
|  | McCauley 2016 | 40 |
|  | McDonald 2019 | 60 |
|  | Norwood 2016 | 60 |
|  | Ovadia 2020 | 20 |
|  | Perrone 2012 | 60 |
|  | Pett 2019 | 80 |
|  | Radomski 2018 | 60 |
|  | Rickles 2021 | 60 |
|  | Rittenhouse 2015 | 60 |
|  | Rutkow 2015 | 80 |
|  | Smith 2015 | 80 |
|  | Thomas 2014 | 20 |
|  | Thornton 2020 | 80 |
|  | Wang 2015 | 40 |
|  | Weiner 2013 | 60 |
|  | Worley 2015 | 100 |
|  | Young 2017 | 20 |

1. The studies were graded out of five criteria worth 20 points each and scored out of 100. Higher scores indicated better methodological quality. [↑](#footnote-ref-1)
